# Enhancing cancer susceptibility to disulfidptosis by inducing cell cycle arrest and impairing DNA repair

**DOI:** 10.7150/thno.122956

**Published:** 2026-01-01

**Authors:** Jing Lin, Xueli Yang, Cizhong Jiang, Xiaoguang Liu, Jiejun Shi

**Affiliations:** 1 Key Laboratory of Spine and Spinal Cord Injury Repair and Regeneration of Ministry of Education, Tongji Hospital affiliated to Tongji University, Frontier Science Center for Stem Cell Research, School of Life Sciences and Technology, Tongji University, Shanghai 200092, China.; 2 Cancer Institute (Key Laboratory of Cancer Prevention and Intervention, China National Ministry of Education) of the Second Affiliated Hospital and Institute of Translational Medicine, Zhejiang University School of Medicine, Hangzhou, China.; 3 Cancer Center of Zhejiang University, Hangzhou, China.; 4 Zhejiang Key Laboratory of Frontier Medical Research on Cancer Metabolism, China.; 5 Shanghai Tenth People's Hospital, Shanghai Key Laboratory of Signaling and Disease Research, School of Life Sciences and Technology, Tongji University, Shanghai 200072, China.

**Keywords:** Disulfidptosis, Cell cycle arrest, DNA repair impairment, PARP inhibitors, Pan-cancer analysis

## Abstract

Disulfidptosis—a regulated cell death caused by disulfide stress under glucose starvation and high SLC7A11—offers a potential cancer vulnerability, but its regulatory landscape and therapeutic tractability remain unclear. We sought to (i) map disulfidptosis susceptibility across cancers, (ii) define associated pathways and regulators, and (iii) test whether targeting these pathways enhances disulfidptosis to improve antitumor efficacy.

**Methods:** We curated 43 core regulators to compute the disulfidptosis score (D-score) across ~10,000 TCGA tumors, benchmarked with glucose-starvation datasets. Correlation screening yielded 506 candidate regulators, integrated into a refined score (D-score+). We associated D-score+ with hallmark pathways, genomic instability and DNA-repair signatures. Experimental validation used glucose-deprivation models, non-reducing immunoblotting and immunofluorescence of cytoskeletal proteins, CRISPR perturbations, and pharmacologic combinations with cell-cycle arrest agents and PARP inhibitors. Public clinical and drug-response cohorts supported translational analyses.

**Results:** D-score tracked experimental triggers (glucose starvation) and revealed cancer-type-specific prognostic patterns. D-score+ positively correlated with cell-cycle programs (e.g., G2/M checkpoint, spindle) and negatively with DNA-repair activity, while aligning with multiple genomic-instability signatures. Beyond F-actin, tubulin exhibited disulfide-dependent mobility shifts and microtubule disassembly. Combining disulfidptosis with cell-cycle arrest drugs synergistically increased cell death across models, with dose-responsive effects and cross-cancer activity. PARP inhibition synergized with disulfidptosis in multiple lines, and higher susceptibility tracked with PARP-inhibitor sensitivity datasets; CRISPR loss of ATM or FANCD2 further sensitized cells. D-score+ was lower in metastatic versus primary tumors and inversely related to EMT in select cancers; glucose starvation impaired migration in wound-healing assays.

**Conclusions:** Inducing cell-cycle arrest and compromising DNA repair enhances cancer susceptibility to disulfidptosis, in part via redox-dependent disruption of actin and microtubules. D-score/D-score+ provide quantitative biomarkers to stratify tumors for combination strategies pairing disulfidptosis induction with cell-cycle inhibitors or PARP inhibitors. These findings nominate disulfidptosis-related pathways as actionable targets and support integrating disulfidptosis profiling into precision oncology, warranting *in vivo* and clinical validation.

## Introduction

Regulated cell death (RCD) is a critical mechanism for maintaining both cellular and metabolic homeostasis. It prevents diseases and supports proper development, immunity, and stress responses 1. Among the various forms of RCD, the recently defined disulfidptosis is uniquely characterized by a profound disruption of protein homeostasis, primarily due to the accumulation of incorrect disulfide bonds 2,3. This process is particularly prevalent under conditions of glucose deprivation and overexpression of the cystine transporter SLC7A11 4. The accumulation of disulfide bonds impairs the proper functioning of cellular proteins, leading to cellular stress, and eventually, cell death 5. This distinguishes disulfidptosis from other oxidative stress-driven forms of cell death 4,6. Understanding disulfidptosis opens new possibilities for cancer treatment. Targeting disulfidptosis may offer a novel therapeutic strategy that exploits the metabolic vulnerabilities of cancer cells, potentially leading to more effective treatments that selectively induce cell death in *SLC7A11*-overexpressing tumors. However, the precise mechanisms and regulatory targets of disulfidptosis remain largely elusive.

One of the consequences of excessive disulfide stress is the collapse of the actin cytoskeleton. The formation of disulfide bonds leads to the contraction of actin filament (F-actin), the polymerized form of actin, disrupting its ability to maintain proper cellular connections 3. This results in a compromised ability to support the plasma membrane and overall cellular architecture. These findings highlight the significant impact of disulfide homeostasis on the cytoskeleton, suggesting it as an emerging hallmark of dysregulated cell death. However, the effects of excessive disulfide stress may extend beyond actin. Whether other pathways are affected remains unknown.

In this study, we compiled 43 experimentally identified promoters and suppressors of disulfidptosis. Through an integrative multi-omics analysis of over 30 cancer types from approximately 10,000 patients, we mapped the disulfidptosis susceptibility across various cancers. Additionally, through co-expression analysis, we identified potential new regulatory genes and pathways involved in disulfidptosis. Our analysis revealed that disulfidptosis susceptibility is significantly associated with the activity of cell cycle and DNA damage repair pathways. Using both disulfidptosis cell models and *in silico* approaches, we confirmed that multiple cell cycle arrest drugs and PARP inhibitors could synergistically work with disulfidptosis to promote tumor cell death. Our findings uncover unprecedented regulatory targets for disulfidptosis and may offer insights into drug resistance and new therapeutic strategies for cancer treatment.

## Results

### Assessing disulfidptosis susceptibility based on core regulator expression

**Figure 1:**
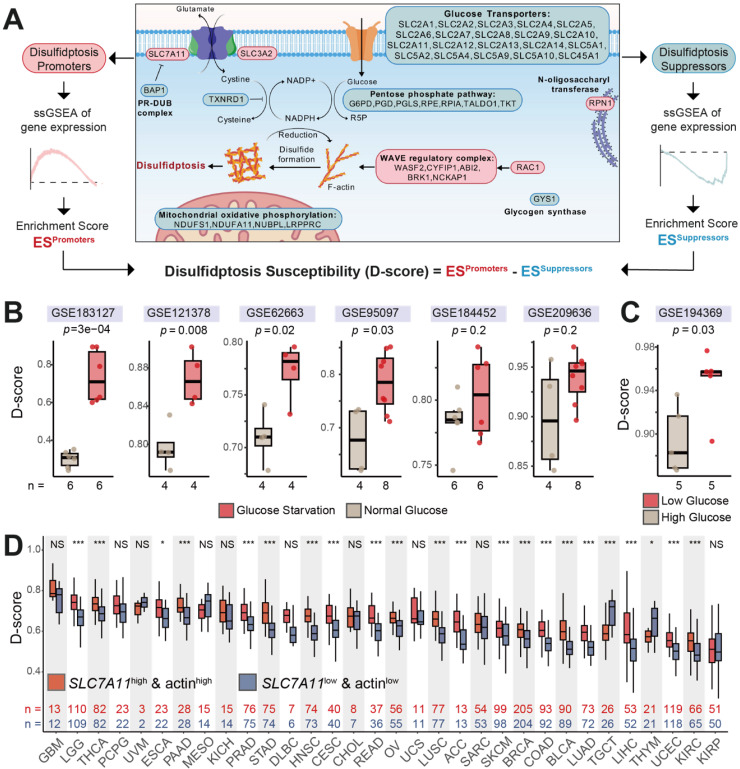
** Assessment of disulfidptosis susceptibility based on the expression of core regulators.** (A) Schematic representation of disulfidptosis promoters (red) and suppressors (blue), and the calculation of the disulfidptosis susceptibility score (D-score). The schematic diagram in the middle was created using BioGDP.com 37. (B) D-scores are significantly higher in glucose starvation-treated samples compared to controls. Sample sizes are indicated below each box. (C) D-scores are significantly lower in high-glucose conditions compared to low-glucose conditions. (D) In most TCGA cancer types, patients with high *SLC7A11* and high actin expression (*SLC7A11*^high^ & actin^high^) exhibit significantly higher D-scores than those with low *SLC7A11* and low actin expression (*SLC7A11*^low^ & actin^low^). Statistical significance was assessed using unpaired two-tailed Wilcoxon test.

Based on previous studies 2,3,7-9, 43 core regulators involved in disulfidptosis were selected for subsequent analysis (Figure 1A and Table S1). These include 9 positive regulators (promoters), such as the cystine transporter SLC7A11 2,3 and its chaperone SLC3A2 3, Rac (RAC1) 3 and WAVE complex-related genes (WASF2, CYFIP1, ABI2, BRK1, NCKAP1) 3 that facilitate lamellipodia formation, and the N-oligosaccharyl transferase RPN1 3. Conversely, 34 negative regulators (suppressors) were also identified, including glucose transporters (SLC2A1 to SLC2A14, SLC5A1, SLC5A2, SLC5A4, SLC5A9, SLC5A10, SLC45A1) 3, pentose phosphate pathway genes (G6PD, PGD, PGLS, RPE, RPIA, TALDO1, TKT) 2,3, glycogen synthase GYS1 3, mitochondrial oxidative phosphorylation genes (NDUFS1, NDUFA11, NUBPL, LRPPRC) 3, deubiquitinase BAP1 which inhibit *SLC7A11* expression 7, and thioredoxin reductase TXNRD1 8,9. Promoters were generally upregulated in tumor tissues compared to their normal counterparts, while suppressors exhibited considerable heterogeneity in their expression profiles (Figure S1A). The prognostic significance of both promoters and suppressors varied substantially across different cancer types (Figure S1B).

To quantify the susceptibility of cells to disulfidptosis, we calculated a disulfidptosis score (D-score) for each sample using the single sample gene set enrichment analysis (ssGSEA) 10 algorithm. The D-score is defined as the difference between the enrichment scores of disulfidptosis promoters and suppressors (Figure 1A). Previous studies have shown that glucose starvation is a prerequisite for cells to undergo disulfidptosis. We thus obtained gene expression data from various cell lines under glucose starvation and high glucose conditions from the GEO database 11-17. The results demonstrated that D-scores were significantly higher in the glucose starvation treatment groups compared to the control groups (Figure 1B), while in the high-glucose treatment group, D-scores were significantly lower than those in the low-glucose group (Figure 1C).

In addition to glucose starvation, high expression of *SLC7A11* is another key requirement for disulfidptosis 3,18. SLC7A11 mediates cystine transport, which accumulates due to an inability to be reduced by NADPH, leading to disulfide bond accumulation and actin cross-linking. This results in cytoskeletal collapse and cell death. Cells with higher actin levels and more branched cytoskeletons, such as those forming lamellipodia, are more susceptible to disulfidptosis under conditions of disulfide bond accumulation 3. To further validate that the D-score accurately reflects disulfidptosis susceptibility, we analyzed gene and protein expression data from TCGA cohorts. Samples from each cancer type were stratified into four groups based on the gene expression levels of *SLC7A11* and the protein expression level of α-actin. D-score comparisons revealed that, in most cancer types, the “*SLC7A11*^high^ & actin^high^” group exhibited significantly higher D-scores than the “*SLC7A11*^low^ & actin^low^” group (Figure 1D). The other two groups (“*SLC7A11*^high^ & actin^low^” and “*SLC7A11*^low^ & actin^high^”) showed intermediate D-scores (Figure S2). These findings suggest that the D-score, based on the 43 core disulfidptosis genes, accurately reflects a cell's susceptibility to undergo disulfidptosis.

### Pan-cancer disulfidptosis susceptibility and its prognostic implications

We next conducted a comprehensively analysis of the distribution of the D-score and its relationship with prognosis across over 10,000 samples from 33 cancer types in the TCGA database. The highest susceptibility was observed in LGG (Low-Grade Glioma), while the lowest was found in LIHC (Liver Hepatocellular Carcinoma). Moreover, with the exception of a few cancer types (e.g., UCEC, UCS, LUAD), most cancers displayed higher disulfidptosis susceptibility in tumor samples compared to their normal counterparts (Figure S3A), suggesting that targeting disulfidptosis could be a promising cancer therapeutic strategy.

We then investigated the relationship between D-score and survival prognosis. After stratifying patients into high and low D-score groups, we employed Cox proportional hazards models to evaluate survival differences between these groups. The hazard ratio comparing the high D-score group with the low D-score group is shown in Figure S3B. In cancer types such as LIHC, BLCA, and KIRC, a high D-score was a significant unfavorable prognostic factor. However, in cancers like THCA, MESO and GBM, a high D-score was significantly associated with a favorable prognosis (Figure S3B). We further analyzed the distribution of D-scores across different clinical stages (Figure S3C). In 14 cancer types (e.g., KICH, CHOL, MESO, SKCM, STAD, UVM, ACC, LIHC, LUAD, KIRP, ESCA, PAAD, KIRC, LUSC), late-stage cancer patients exhibited higher disulfidptosis susceptibility compared to early-stage cancer patients. Conversely, in other cancer types (e.g., THCA, COAD, HNSC, BLCA, READ, BRCA), this trend was reversed. This inter-cancer variation in the relationship between D-score and clinical phenotypes suggests that the impact of disulfidptosis susceptibility on prognosis is cancer type-dependent.

### Identification and characterization of potential disulfidptosis regulators

To identify genes with consistent regulatory roles in disulfidptosis across pan-cancer types, we performed a correlation-based screening using gene expression data from TCGA (Table S2). Genes exhibiting a significant positive correlation with the D-score (adjusted p-value < 0.05) in at least one-third (>10) of cancer types were classified as potential positive regulators of disulfidptosis, referred to as candidate promoters (n = 475). Conversely, genes with a significant negative correlation with the D-score (adjusted p-value < 0.05) in at least one-third of cancer types were classified as potential negative regulators, designated as candidate suppressors (n = 31). Gene Ontology (GO) functional clustering analysis of candidate promoters highlighted pathways related to the cell cycle, microtubule cytoskeleton, and DNA repair (left panel in Figure S4A), while candidate suppressors were enriched for mitochondrial functions and oxidative phosphorylation (right panel in Figure S4A).

**Figure 2:**
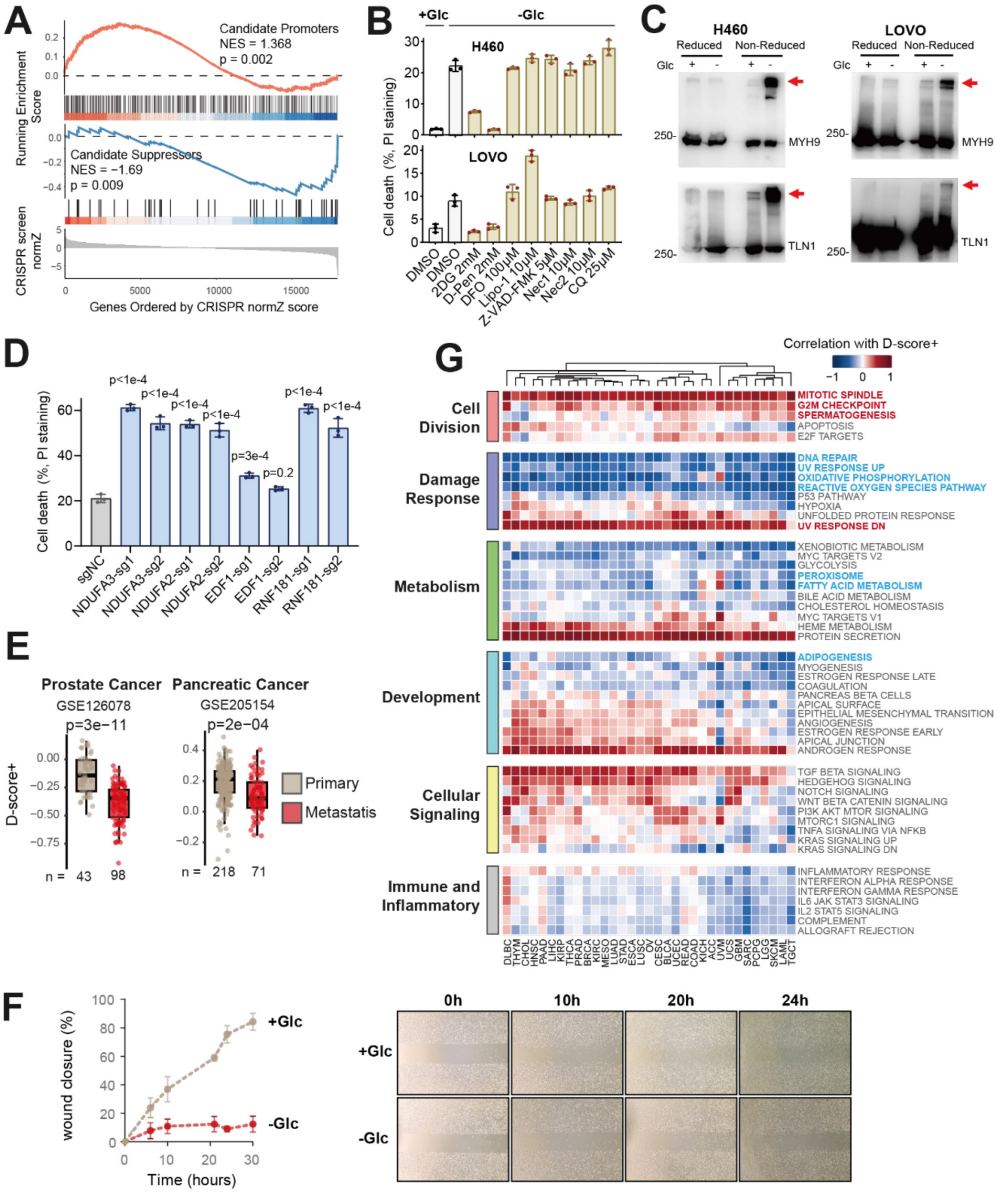
** Identification and Characterization of Potential Disulfidptosis Regulators.** (A) Validation of candidate promoters and suppressors using CRISPR screening data. Genes are ordered by their normZ score, with higher values indicating promotion of disulfidptosis and lower values indicating inhibition. NES (normalized enrichment score) and p-value are calculated by ssGSEA. (B) The glucose deprivation-induced cell death could be suppressed by inhibitors of disulfidptosis but not the other forms of cell death. Inhibitors of disulfidptosis: 2-Deoxy-D-glucose (2DG) and D-penicillamine (D-Pen), ferroptosis: Deferoxamine mesylate (DFO) and Liproxstatin-1 (Lipo-1), apoptosis: Z-VAD-FMK, necroptosis: Necrostatin-1 (Nec1), necrosis: Necrox-2 (Nec2), and autophagy: chloroquine (CQ). (C) The reduced and non-reduced Western blot shows that MYH9 and TLN1 are impaired in the glucose deprivation (-Glc) induced disulfidptosis cell model but not in normal condition (+Glc). (D) CRISPR-Cas9 knockout of top candidate suppressors (i.e., NDUFA3, NDUFA2, EDF1, and RNF181) leads to enhanced cell death of H460 cells in the glucose deprivation (-Glc) condition. Statistical significance was assessed using unpaired two-tailed Student's t-test. (E) Comparison of D-score+ between primary and metastatic tumor samples from prostate (GSE126078) and pancreatic (GSE205154) cancer patients. Statistical significance was assessed using unpaired two-tailed Student's t-test. (F) Scratch-wound assays of LOVO cells cultured with glucose (+Glc) or under glucose deprivation (-Glc). Left, % wound closure over time; right, representative fields at indicated times. (G) Spearman's correlation between D-score+ and the activity of hallmark pathways.

In our previous work, we conducted a whole-genome CRISPR-Cas9 screen under conditions inducing disulfidptosis and assessed the regulatory effects of genes using normZ scores 3. A positive normZ value indicates promotion of disulfidptosis, while a negative value suggests inhibition. Gene Set Enrichment Analysis (GSEA) revealed that candidate promoters significantly promote disulfidptosis (NES = 1.368, p = 0.002, red curve in Figure 2A), while candidate suppressors significantly inhibit it (NES = -1.69, p = 0.009, blue curve in Figure 2A), thus confirming the validity of our correlation-based selection of candidate disulfidptosis regulators.

Based on our previous work in the UMRC6 cell line 3, we established a glucose starvation-induced disulfidptosis model in lung cancer (H460) and colorectal cancer (LOVO) cell lines. To exclude interference from other forms of cell death, we initially treated cells with various cell death inhibitors. Only the disulfidptosis inhibitors 2-Deoxy-D-glucose (2DG) and D-penicillamine (D-Pen) effectively suppressed glucose deprivation-induced cell death. In contrast, inhibitors of ferroptosis (Deferoxamine mesylate and Liproxstatin-1), apoptosis (Z-VAD-FMK), necroptosis (Necrostatin-1), necrosis (Necrox-2), and autophagy (chloroquine) failed to prevent cell death under glucose starvation (Figure 2B). Given that F-actin contraction is a hallmark of disulfidptosis, we next examined whether the function of F-actin-associated proteins was disrupted in our models. Western blotting under non-reducing conditions revealed significant slower electrophoretic mobility of MYH9 and TLN1 after glucose withdrawal, accompanied by the formation of protein condensates (red arrows in Figure 2C). These findings suggest that MYH9 and TLN1 were disrupted by disulfide bonds, impairing their function. Together, these results validate the successful establishment of disulfidptosis cell models.

We then leveraged these models to investigate the regulatory roles of candidate genes identified in our prior analyses. Using CRISPR-Cas9-mediated gene knockout guided by specific sgRNAs, we individually ablated several top-ranked candidate suppressors. Compared to control cells, knockout of these genes led to a marked increase in cell death under glucose starvation (Figure 2D), confirming their role as negative regulators of disulfidptosis and further supporting the validity of our screening results. However, it should be noted that the functional roles of the majority of these candidate regulators remain to be experimentally validated and warrant further investigation.

After integrating these candidate regulators (n = 506) with the known core regulators (n = 43), we developed an enhanced version of the disulfidptosis susceptibility score, referred to as D-score+. Previous studies in kidney cancer cell lines have shown that disulfidptosis contributes to tumor metastasis inhibition 18. Our analysis on two datasets with large sample sizes of metastatic cancer validated that D-score+ is significantly lower in metastatic cancer than primary cancer (Figure 2E). In addition, we utilized metastasis status information from TCGA (Mstage) to divide patient groups and compared their D-score+ values. In 6 of the 8 cancer types with both M0 (without distant metastasis) and M1 (with distant metastasis) patients, the D-score+ was consistently lower in M1 patients compared to M0 patients (Figure S4B). Consistently, wound healing assays in LOVO cells revealed that glucose starvation (-Glc), a condition that induces disulfidptosis, markedly impaired migratory capacity compared with normal conditions (+Glc), supporting a direct role of disulfidptosis in suppressing metastatic potential (Figure 2F). These findings suggest that disulfidptosis inhibits metastasis in most cancer types, thereby further validating the plausibility of D-score+.

In addition to Mstage, we assessed the relationship between D-score+ and the activity of the hallmark epithelial-mesenchymal transition (EMT) pathway in the TCGA pan-cancer dataset. Consistent with previous findings in kidney cancer cell lines 18, D-score+ was negatively correlated with EMT scores in KIRP and KICH, suggesting that disulfidptosis inhibits metastasis in these tumors (Figure S4C). However, the correlation varies from -0.56 to 0.69 across different cancer types and only exhibits significance in 23 of them (p<0.05). These analyses further reveal the heterogeneity of disulfidptosis's impact on EMT activity across different cancer types.

Next, we aimed to identify cell pathways consistently associated with disulfidptosis susceptibility. We collected 50 hallmark gene sets from MSigDB and used ssGSEA to score each TCGA sample, calculating the correlation between each hallmark pathway score and D-score+ (Figure 2G). Consistent with prior studies, D-score+ exhibited a pan-cancer negative correlation with pathways related to fatty acid metabolism, peroxisome, and adipogenesis ('Metabolism' and 'Development' modules in Figure 2G). Lipid peroxidation, leading to membrane damage, is a hallmark of another form of cell death—ferroptosis 19,20. However, ferroptosis and disulfidptosis have opposite triggering conditions: ferroptosis is associated with low *SLC7A11* expression, whereas disulfidptosis correlates with high *SLC7A11* expression 4,21. These findings highlight distinct regulatory patterns between the two forms of cell death and further validate the relevance of D-score+ as a measure of disulfidptosis susceptibility.

Interestingly, we also found that D-score+ was significantly positively correlated with cell cycle-related pathways, such as the G2M checkpoint, mitotic spindle, and spermatogenesis, across various cancers ('Cell Division' module in Figure 2G). This suggests a potential link between the onset of disulfidptosis and cell cycle regulation. In addition, D-score+ showed a consistent negative correlation with the activities of the hallmarks of “DNA Repair” and “UV Response Up” ('Damage Response' module in Figure 2G), suggesting that disulfidptosis may be associated with impaired DNA damage repair efficacy.

Altogether, these results demonstrate that disulfidptosis is broadly connected to hallmark pathways, such as cell cycle regulation and DNA repair.

### Synergistic effects of cell cycle arrest drugs and disulfidptosis

**Figure 3:**
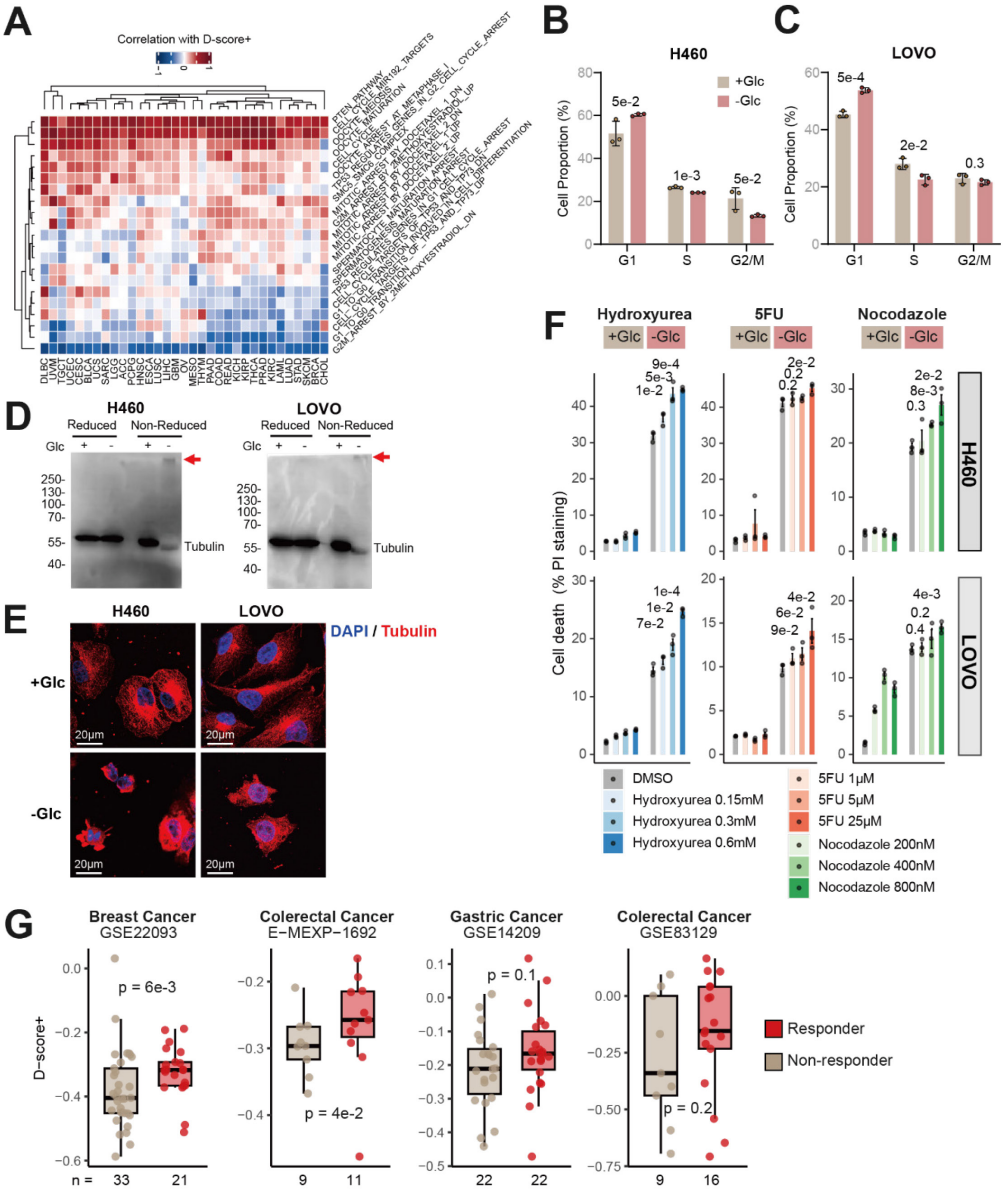
** Synergistic Effects of Cell Cycle Arrest Drugs and Disulfidptosis.** (A) Correlation between D-score+ and activities of cell-cycle related pathways. (B-C) Cell cycle distribution analysis in H460 and LOVO cells undergoing disulfidptosis induction (-Glc). Cell proportion was quantified in three replicates. Statistical significance between untreated (DMSO) and treated samples of each drug concentration was assessed using unpaired two-tailed Student's t-test. (D) The reduced and non-reduced Western blot shows that tubulin structure is impaired in the glucose deprivation (-Glc) induced disulfidptosis cell model but not in normal condition (+Glc). (E) Fluorescence staining of tubulin in H460 and LOVO cells under normal glucose (+Glc) and glucose deprivation (-Glc) conditions. (F) Cell cycle arrest drugs enhance cell death in disulfidptosis cell models. Three replicates of H460 (upper panel) and LOVO cells (lower panel) were treated with the drugs for 3 and 9 hours, respectively, followed by quantification of cell death. Statistical significance between untreated (DMSO) and treated samples of each drug concentration was assessed using unpaired one-tailed Student's t-test. (G) Higher disulfidptosis susceptibility in patients responding to cell cycle arrest drug treatment. Statistical significance was assessed using unpaired two-tailed Student's t-test.

We examined cell cycle-related pathways from multiple databases (GO, KEGG, BIOCARTA) and found that D-score+ was consistently correlated with multiple cell-cycle arrest-related terms across cancers (Figure 3A). Then, we performed cell cycle analysis in H460 and LOVO cells under disulfidptosis conditions (-Glc). Quantitative results revealed a significant increase in the G1-phase cell population compared to controls, along with decreasing trends in both S-phase and G2/M-phase distributions (Fig 3B and C). These data provide direct experimental evidence that disulfidptosis induces functional alterations in the cell cycle and prompted us to investigate whether cell cycle-related proteins are affected under disulfide stress.

Our previous analysis found that disulfidptosis is consistently correlated with mitotic spindle (Figure 2G). Therefore, we first checked the disulfidptosis relevance of tubulin, which plays key roles in spindle assembly. Analysis of gene and protein expression data from TCGA revealed that patients classified as “*SLC7A11*^high^ & tubulin^high^” exhibited higher disulfidptosis susceptibility compared to “*SLC7A11*^low^ & tubulin^low^” patients (Figure S5), suggesting that tubulin may be impacted during disulfidptosis. In both H460 and LOVO cell models, after glucose starvation (-Glc) and before significant cell death was observed, we observed a significant alteration in the electrophoretic mobility of tubulin under non-reducing conditions, resulting in the formation of protein condensates larger than 250 kDa (red arrows in Figure 3D). Meanwhile, fluorescence staining further demonstrated that microtubule structures were disrupted under glucose starvation (-Glc) conditions, leading to a loss of normal cell morphology (Figure 3E). These findings support our conclusion that, in addition to actin, microtubules represent another target of disulfide bond-mediated damage during disulfidptosis.

We next explored whether inducing disulfidptosis in combination with cell cycle arrest drugs could synergistically promote cell death. To address this, we conducted *in vitro* experiments to evaluate the impact of drug treatment on disulfidptosis. As shown in Figure 3F, under glucose-sufficient conditions (+Glc), we observed the effects of individual drugs on cell viability. Under glucose starvation conditions (-Glc), we then assessed the synergistic impact of drug treatment combined with disulfidptosis on cell survival. Cell cycle arrest agents such as Hydroxyurea, 5-Fluorouracil (5-FU), and Nocodazole, significantly enhanced cell death when combined with disulfidptosis in both H460 and LOVO cells, demonstrating notable synergistic effects (Figure 3F). To further validate the cross-cancer applicability of our findings, we expanded our experiments to include KYSE-150, an esophageal squamous cell carcinoma cell line. This selection was based on our computational analysis, which indicated high disulfidptosis susceptibility in esophageal carcinoma (ESCA in Figure S3A). Our results show significant synergy between disulfidptosis induction and cell cycle inhibitors in KYSE-150 cells (Figure S6). Moreover, in all three cell models, the synergistic effect was enhanced with increasing drug concentrations, which suggest that the optimal drug concentration may still have room for further optimization.

To further validate the synergistic effects of cell cycle arrest drugs with disulfidptosis at the patient level, we analyzed multiple drug treatment cohorts from the GEO and ArrayExpress databases (GSE22093, E-MEXP-1692, GSE14209, GSE83129) 22-25. We examined the relationship between drug efficacy and disulfidptosis susceptibility. As illustrated in Figure 3G, in breast cancer cohorts, responders to 5-FU treatment exhibited significantly higher disulfidptosis susceptibility compared to non-responders. Similarly, in colorectal and gastric cancer cohorts, responders also demonstrated higher disulfidptosis susceptibility scores. However, due to limited sample sizes, statistical significance was not reached in two of the cohorts.

In summary, the results from clinical cohorts align with cellular experiments, demonstrating that tubulin represents a distinct case of non-actin proteins being impaired by disulfide bonds, and cell cycle arrest drugs can synergize with disulfidptosis. These findings underscore the potential of combining cell cycle arrest agents with disulfidptosis-inducing therapies as a novel strategy for cancer treatment.

### Disulfidptosis as a biomarker for PARP inhibitor sensitivity

**Figure 4:**
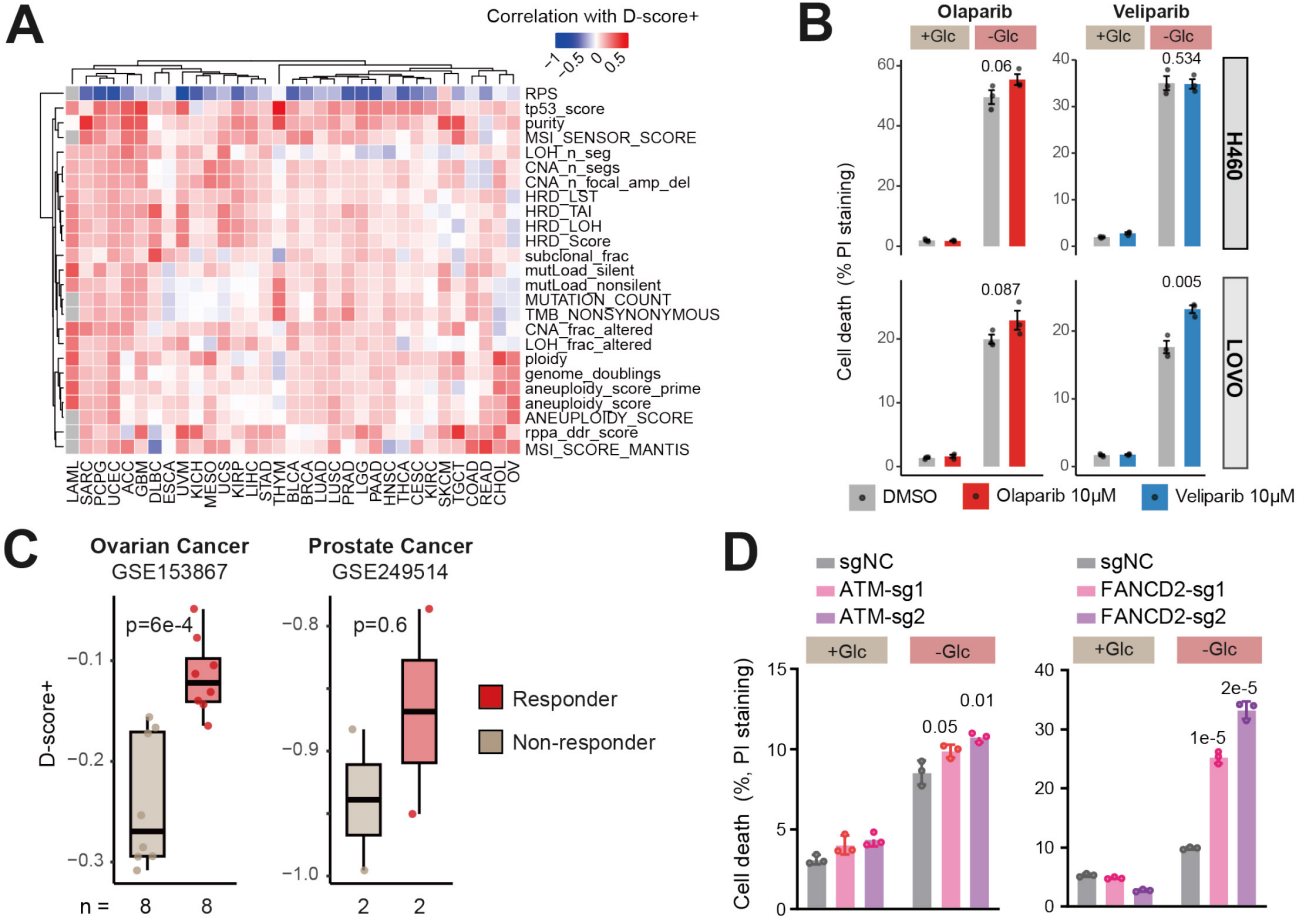
** Disulfidptosis susceptibility facilitates PARP inhibitor sensitivity.** (A) Spearman's correlation between D-score+ and 25 genomic aberration signatures. (B) Synergistic effects between disulfidptosis and PARP inhibitors in H460 cells (upper panel) and LOVO cells (lower panel). Three replicates of H460 and LOVO cells were treated with the drugs for 6 and 14 hours, respectively, followed by quantification of cell death. Statistical significance between untreated (DMSO) and treated samples was assessed using unpaired one-tailed Student's t-test. (C) Higher disulfidptosis susceptibility in responders to PARP inhibitor treatment. Statistical significance was assessed using unpaired two-tailed Student's t-test. (D) LOVO cells expressing control sgRNA (sgNC) or sgRNAs targeting *ATM* (left) or *FANCD2* (right) were cultured in glucose-replete (+Glc) or glucose-starved (-Glc) conditions. Cell death was measured in 3 replicates. Statistical significance was assessed using unpaired one-tailed Student's t-test.

Our earlier findings in the TCGA cohort revealed that disulfidptosis susceptibility is significantly associated with DNA damage repair-related hallmarks, such as "DNA_Repair," "UV_Response_DN," and "UV_Response_UP," in numerous cancer types (Figure 2G). To explore the relationship between disulfidptosis and genome instability, we correlated D-score+ with 25 genomic aberration signatures collected from the GDC portal across different cancer types. Among these signatures, only one was related to DNA repair efficacy—recombination proficiency score (RPS) 26—while the others were related to genome instability, such as loss of heterozygosity (LOH), microsatellite instability (MSI), homologous recombination deficiency (HRD), etc. Our results indicate that D-score+ is consistently negatively correlated with RPS but positively correlated with genome instability signatures (Figure 4A), suggesting that disulfidptosis is linked to genome instability and may influence DNA damage repair processes. To explore this further, we investigated whether DNA damage repair-targeting drugs, specifically PARP inhibitors (PARPi), could synergize with disulfidptosis to enhance cell death.

Using the glucose starvation-induced disulfidptosis model in H460 and LOVO cell lines, we treated the cells with PARP inhibitors, including Olaparib and Veliparib. In LOVO cells (lower panel in Figure 4B), we observed synergistic effects between disulfidptosis and both PARP inhibitors. However, in H460 cells, only the combination with Olaparib showed a synergistic effect, while Veliparib did not (upper panel in Figure 4B). This discrepancy may be attributed to off-target effects of the drugs or to cell-type-specific differences in the synergistic effect. Therefore, in addition to NCI-H460, we developed another lung cancer disulfidptosis model in NCI-H226 cells and observed significant synergy between veliparib and disulfidptosis induction (Figure S7). Although the precise mechanism remains unclear, these findings underscore the importance of sample-specific sensitivity in combination therapies.

Additionally, we analyzed publicly available data containing gene expression profiles and phenotypic data from PARP inhibitor treatments. As shown in Figure 4C, analysis of the GSE153867 dataset, which includes Olaparib-treated ovarian cancer cell lines 27, D-scores were significantly lower in resistant samples compared to non-resistant samples. Similarly, in GSE249514, which includes Olaparib-treated castration-sensitive prostate cancer (CSPC) cell lines 28, we observed the same trend, although statistical significance was not reached due to limited sample size. To further explore the functional link, we performed CRISPR-mediated knockdown of DNA damage repair genes. Loss of *ATM* or *FANCD2* significantly enhanced glucose starvation-induced cell death, supporting their role in protecting against disulfidptosis (Figure 4D). Knockdown of *RAD51* produced a similar trend; however, only one sgRNA (RAD51-sg2) yield a significant increase in cell death. This discrepancy may reflect differences in sgRNA efficiency or partial compensation by residual RAD51 activity (Figure S8). These results indicate that samples with higher disulfidptosis susceptibility are more sensitive to PARP inhibitors.

Overall, our findings suggest that disulfidptosis susceptibility could serve as a novel biomarker to guide PARP inhibitor treatment, complementing existing molecular markers and potentially improving patient stratification in precision oncology.

## Discussion

Disulfidptosis is a newly characterized form of regulated cell death caused by disulfide stress—a cytotoxic subtype of oxidative stress 29. Our previous work first demonstrated that excessive disulfide bond accumulation disrupts redox homeostasis and damages F-actin, leading to cell death 2,3. Building upon these, the present study comprehensively curated 43 core regulators of disulfidptosis, enabling a more accurate assessment of cellular susceptibility to this process.

This study addresses the limitations of previous studies, which defined disulfidptosis-related genes in a largely correlative and incomplete manner. For instance, Zhang et al. proposed 16 signature genes 30, but only *SLC7A11* is functionally implicated in disulfidptosis regulation. Similarly, Zhao et al. included 23 genes 31, of which some, such as *ATF4* and *PRC1*, lack direct evidence for involvement in disulfidptosis. More critically, they failed to include glucose transporters (*SLC2A, SLC5A* families and* SLC45A1*), and key pentose phosphate pathway genes (*PGLS, RPE, RPIA*), all of which are crucial for maintaining redox balance via NADPH production. As a result, susceptibility based on these incomplete gene sets likely overlooked potential regulatory axes of disulfidptosis.

We developed the disulfidptosis score (D-score) and its refined variant (D-score+) to quantify this susceptibility. Through comparisons between glucose deprivation and normal samples and analysis with *SLC7A11* expression and actin protein levels, we confirmed that these metrics reliably captured disulfidptosis vulnerability and showed promise in stratifying cancer types. Additionally, higher D-scores were consistently associated with reduced metastatic potential, suggesting therapeutic opportunities to suppress metastasis via disulfidptosis induction.

A key insight of this study is the discovery of a mechanistic link between disulfidptosis and cell cycle regulation. Tubulin, a core cytoskeletal and mitotic component, was identified as a novel target of disulfide stress, distinct from the known F-actin disruption. Inducing cell cycle arrest using hydroxyurea, 5-FU, or nocodazole markedly enhanced disulfidptosis-induced cell death, though the degree of synergy varied across cell lines. This redox-cell cycle interaction opens new avenues for combination therapy design.

In parallel, we found that high disulfidptosis susceptibility correlates with impaired DNA repair activity. PARP inhibitors (Olaparib, Veliparib) exhibited enhanced cytotoxicity in high-D-score cells, especially in LOVO cells, though the response in H460 cells was modest, indicating cell-context-specific differences.

Despite these insights, several limitations should be acknowledged. First, mechanistic details, such as how tubulin undergoes disulfide modification or how DNA repair dysfunction amplifies disulfidptosis, remain unclear. Current work does not pinpoint the specific cysteine residues involved in disulfide bond formation on tubulin, which would require redox mass spectrometry in combination with site-directed mutagenesis. As tubulin is essential for cell growth and proliferation, mutational analysis is not feasible in our system and should be explored in future. Second, our findings are based primarily on cell lines and computational analyses, warranting further validation *in vivo* and in patient-derived models. We have consulted with clinical collaborators to explore the feasibility of patient recruitment. However, as disulfidptosis is a newly identified and relatively underexplored form of cell death, patient willingness to participate is currently very limited, making prospective validation unfeasible at this stage. Third, the heterogeneous responses across different cell lines highlight the influence of tumor-specific redox and metabolic contexts, suggesting that disulfidptosis-targeted strategies may require patient stratification. Last, as publicly available transcriptomic data from monotherapy PARP inhibitor treatments are scarce, large-scale patient-derived xenograft (PDX) model may be useful to further validate its clinical relevance, which is currently beyond our available resources.

Nevertheless, this study establishes a robust framework for characterizing disulfidptosis susceptibility, uncovers novel mechanistic links with the cell cycle and DNA repair, and suggests new opportunities for combination therapies. Future studies should aim to define the molecular circuitry of disulfidptosis in greater detail and validate its therapeutic potential in preclinical and clinical settings. Ultimately, integrating disulfidptosis profiling into precision oncology may enable the development of personalized redox-targeted interventions for cancer treatment.

## Methods

### Disulfidptosis susceptibility measurement

We identified 43 core regulators related to disulfidptosis based on our previous studies 2,3, including 9 promoters (SLC7A11, SLC3A2, RAC1, WASF2, CYFIP1, ABI2, BRK1, NCKAP1, RPN1) and 34 suppressors (SLC2A1, SLC2A2, SLC2A3, SLC2A4, SLC2A5, SLC2A6, SLC2A7, SLC2A8, SLC2A9, SLC2A10, SLC2A11, SLC2A12, SLC2A13, SLC2A14, SLC5A1, SLC5A2, SLC5A4, SLC5A9, SLC5A10, SLC45A1, G6PD, PGD, PGLS, RPE, RPIA, TALDO1, TKT, GYS1, NDUFS1, NDUFA11, NUBPL, LRPPRC, BAP1, TXNRD1). We used the R package GSVA 10 for single-sample gene set enrichment analysis (ssGSEA) to calculate the enrichment scores (ES) of the promoter and suppressor gene sets. Disulfidptosis susceptibility(D-score) was defined as the difference between the promoter ES and suppressor ES.

For the enhanced version of the D-score, we first performed a pan-cancer correlation screening to identify candidate disulfidptosis regulators. Genes exhibiting a correlation greater than 0.45 with the D-score and an adjusted p-value below 0.05 in at least one-third (>10) of cancer types were classified as candidate promoters. Genes with a correlation below -0.45 and an adjusted p-value below 0.05 in at least one-third of cancer types were classified as candidate suppressors. The lists of candidate promoters and suppressors are provided in Table S2. We then integrated these candidate regulators with the core regulators identified previously and developed the refined disulfidptosis score (D-score+) using the same approach as for D-score.

### Data collection

Gene and protein expression data for tumor tissues, including 33 cancer types from the TCGA dataset, and normal tissue gene expression data from the GTEx dataset were downloaded from the UCSC Xena website (https://xenabrowser.net/datapages/). Phenotypic information for the tumor tissue samples, including overall survival, metastasis status (Mstage), and other clinical variables, was also obtained from UCSC Xena. Additionally, gene expression data from glucose starvation-treated samples and corresponding control samples (GSE183127, GSE121378, GSE62663, GSE95097, GSE184452, GSE209636 and GSE194369) 11-17 were collected from the GEO database to validate the feasibility of disulfidptosis susceptibility. We also retrieved several datasets from the ArrayExpress and GEO databases to examine the correlation between disulfidptosis susceptibility and responses to cell cycle arrest drugs (E-MEXP-1692, GSE22093, GSE14209, and GSE83129) 22-25 and PARP inhibitors (GSE249514 and GSE153867) 27,28.

### Correlation between disulfidptosis susceptibility and hallmark activities

Hallmark gene sets and cell cycle-related gene sets were collected from the MSigDB website (https://www.gsea-msigdb.org/gsea/msigdb/) for gene set enrichment analysis (GSEA) 32. Then we conducted ssGSEA using the “GSVA” R package to calculate the enrichment scores (ES) for 50 hallmark pathways curated by MSigDB 33 across 33 cancer types in the TCGA dataset. Spearman correlation was then used to determine the relationship between the ES of these pathways and the D-score+.

### EMT score estimation

To examine the correlation between disulfidptosis and tumor metastasis, we estimated the epithelial-mesenchymal transition (EMT) score based on previous studies 34. Seventy-six metastatic-related tumor genes identified in this study were analyzed using principal component analysis (PCA). The first principal component score was used as the EMT score.

### Gene pathway enrichment analysis

Gene Ontology (GO) functional clustering analysis of the disulfidptosis candidate genes was performed using the clusterProfiler R package 35. Significant terms were defined by an adjusted p-value < 0.05.

### Genomic aberration signature analysis

The 25 genomic aberration signatures across TCGA pan-cancer were collected from the GDC portal (https://gdc.cancer.gov/about-data/publications/pancanatlas), which is curated by previous studies 36. These include RPS (recombination proficiency score), TMB (tumor mutation burden), LOH (loss of heterozygosity), MSI (microsatellite instability), aneuploidy score, genome doubling, TAI (Telomeric Allelic Imbalance), LST (large-scale state transitions), HRD (homologous recombination deficiency), CNA (copy number alteration), and others. Spearman's correlations were calculated between each signature and D-score+ in each cancer types, and hierarchical clustering was performed.

### Survival analysis

To assess the relationship between D-score and patient survival, we performed Kaplan-Meier survival analysis. Patients were divided into high and low D-score groups based on the median D-score. Survival differences were evaluated using the Log-rank test, and Kaplan-Meier curves were generated. We further used Cox proportional hazards regression, with D-score as a continuous variable and clinical covariates (e.g., age, gender, tumor stage) included in the model. Hazard ratios (HR) and 95% confidence intervals (CI) were calculated, with statistical significance determined by the Wald test (p < 0.05). All analyses were conducted using the “surviminer” (https://github.com/kassambara/survminer) and “survival” (https://github.com/therneau/survival) R packages.

### Cell culture and treatment

All cells were obtained from the Cell Bank in the Chinese Academy of Sciences. All these cells were maintained in PRMI-1640 supplemented with 10% fetal bovine serum (Lonsera) and 1% penicillin-streptomycin. All cell lines were free of Mycoplasma contamination (tested by the vendor). None of the cell lines used in this study have been found in the International Cell Line Authentication Committee database of commonly misidentified cell lines, based on short tandem repeat profiling performed by the vendor. For the glucose deprivation experiments, cells were cultured in glucose-free medium with dialyzed fetal bovine serum (Lonsera) with/without indicated chemical treatments. Deferoxamine mesylate was obtained from MCE (HY-B0988). Liproxstatin-1 was obtained from Aladdin (L413818). Z-VAD-FMK was obtained from MCE (HY-16658B). Necrostatin-1 was obtained from MCE (HY-15760). Necrox-2 was obtained from Aladdin (N386066). Chloroquine was obtained from MCE (HY-17589A). 2-Deoxy-D-glucose was obtained from MCE (HY-13966). D-penicillamine was obtained from Sigma-Aldrich (P4875). Hydroxyurea (S1961), Nocodazole (S1765) and Veliparib (SC0020) were obtained from Beyotime. 5-Fluorouracil (5-FU) (HY-90006) was obtained from MCE. Olaparib (GC17580) was obtained from Glpbio.

### Cell death assays

Cell death was measured as described previously 3. Cells were seeded in 24-well plates one day before treatment. After treatment, the cells were trypsinized and collected in 1.5-mL microtubes, washed and resuspended in 1 μg/mL propidium iodide (PI) in pre-cold PBS. The PI-positive (dead) cells were analyzed by a follow cytometer (FongCyte, Challenbio).

### Western blotting

Western blotting was conducted as previously described 3. Briefly, cells in 6 cm dish were harvested and lysed in NP40 buffer followed by centrifugation. The supernatant was combined with loading buffer without reducing agents and split into two aliquots per sample. One aliquot was for non-reducing analysis and β-mercaptoethanol was added to one aliquot for reducing analysis. All samples were incubated at 70 °C for 10 min before SDS-PAGE analysis. Tubulin antibody from Proteintech was used for western blotting.

### Stable cell line generation and CRISPR-Cas9-mediated gene knockout

HEK293T cells were transfected with LentiCRISPR-V2 lentiviral constructs together with the psPAX.2 and pMD2.G packaging plasmids using polyethylenimine (PEI) reagent. After 72 h, the supernatants containing lentiviral particles were collected and filtered through a 0.45 μm membrane. Target cell lines were then infected with the lentivirus in the presence of polybrene (8 μg/mL). Following a 24-h incubation, the medium was replaced with fresh medium containing puromycin (5 μg/mL) for 1-2 weeks of selection, yielding stable cell lines with successful transduction. The gRNA target sequences were as follows: NDUFA3-sg1, 5'-GTACTCCGTCATGATCAACA-3'; NDUFA3-sg2, 5'-CGGGCATGTTCCCATCATCA-3'; NDUFA2-sg1, 5'-CTTCATTGAGAAACGCTACG-3'; NDUFA2-sg2, 5'-AGTGGATGCGAATCTCACGC-3'; EDF1-sg1, 5'-GTGATCGCGGACTATGAGAG-3'; EDF1-sg2, 5'-CCATGACAGGGTGACCCTGG-3'; RNF181-sg1, 5'-CAAATTCCAAAAGACACACG-3'; RNF181-sg2, 5'-AGCCTCTGATGACTGTCCTG-3'; ATM-sg1, 5'-GTGAAATATCTCAGCAACAG-3'; ATM-sg2, 5'-CAGCCTCAACACAAGCCTCC-3'; FANCD2-sg1, 5'-AGAAGCTCTTTCAGACCCTG-3'; FANCD2-sg2, 5'-ATAGGAAGTTTGGGTCAAGT-3'; RAD51-sg1, 5'-GCCATGTACATTGACACTGA-3'; RAD51-sg2, 5'-AGCTGGATTCCATACTGTGG-3'.

### Fluorescence staining of tubulin

Following glucose starvation treatment, cells seeded on glass coverslips were washed with PBS twice and fixed with 4% paraformaldehyde for 10 min. Afterwards, 0.5% Triton X-100 was used to permeate the membrane. After blocked with 5% bovine serum albumin, cells were incubated with tubulin antibody (1:500, Proteintech) overnight at 4°C and a secondary antibody for one hour at room temperature. After stained with DAPI, coverslips were mounted on glass slides with mounting solution (F4680, Sigma). Images were acquired with a confocal microscope (TCS SP8, Leica).

### Wound healing assays

Cells were seeded in 12-well plates one day before wounded with a p20 pipette tip. After replaced with glucose-free medium, the cells were imaged overtime using a microscope. The percentage of wound closure was measured and calculated with ImageJ.

### Statistical analysis

All data analyses were performed using R (version 4.3.3). The Wilcoxon rank-sum test was applied to compare differences between two groups, and the Chi-squared test was used for comparisons among three or more groups. Log-rank tests were used to assess the significance of survival differences between groups. The correspondence between p-value symbols and their numerical ranges is as follows: NS, P ≥ 0.05; *, P < 0.05; **, P < 0.01; ***, P < 0.001.

## Supplementary Material

Supplementary figures and table legends.

Supplementary table 1.

Supplementary table 2.

## Abbreviations

RCD: regulated cell death
ssGSEA: single-sample gene set enrichment analysis
GSEA: Gene Set Enrichment Analysis
GSVA: Gene Set Variation Analysis
GO: Gene Ontology
KEGG: Kyoto Encyclopedia of Genes and Genomes
BIOCARTA: BioCarta pathway database
EMT: epithelial-mesenchymal transition
PARP: poly(ADP-ribose) polymerase
PARPi: PARP inhibitor
RPS: recombination proficiency score
TMB: tumor mutation burden
LOH: loss of heterozygosity
MSI: microsatellite instability
HRD: homologous recombination deficiency
TAI: telomeric allelic imbalance
LST: large-scale state transitions
CNA: copy-number alteration
CRISPR: clustered regularly interspaced short palindromic repeats
Cas9: CRISPR-associated protein 9
sgRNA: single guide RNA
PCA: principal component analysis
ES: enrichment score
NES: normalized enrichment score
HR: hazard ratio
CI: confidence interval
PDX: patient-derived xenograft
RPMI-1640: Roswell Park Memorial Institute 1640 medium
F-actin: filamentous actin
PI: propidium iodide
PBS: phosphate-buffered saline
NP-40: Nonidet P-40
SDS-PAGE: sodium dodecyl sulfate-polyacrylamide gel electrophoresis
DAPI: 4′,6-diamidino-2-phenylindole
PEI: polyethylenimine
2DG: 2-deoxy-D-glucose
D-Pen: D-penicillamine
Z-VAD-FMK: Z-Val-Ala-Asp(OMe)-fluoromethyl ketone
Glc: glucose
UV: ultraviolet
NADPH: nicotinamide adenine dinucleotide phosphate (reduced form)
D-score: disulfidptosis score
D-score+: refined disulfidptosis susceptibility score
LGG: Lower Grade Glioma
LIHC: Liver Hepatocellular Carcinoma
UCEC: Uterine Corpus Endometrial Carcinoma
UCS: Uterine Carcinosarcoma
LUAD: Lung Adenocarcinoma
KICH: Kidney Chromophobe
CHOL: Cholangiocarcinoma
MESO: Mesothelioma
SKCM: Skin Cutaneous Melanoma
STAD: Stomach Adenocarcinoma
UVM: Uveal Melanoma
ACC: Adrenocortical Carcinoma
KIRP: Kidney Renal Papillary Cell Carcinoma
ESCA: Esophageal Carcinoma
PAAD: Pancreatic Adenocarcinoma
KIRC: Kidney Renal Clear Cell Carcinoma
LUSC: Lung Squamous Cell Carcinoma
THCA: Thyroid Carcinoma
COAD: Colon Adenocarcinoma
HNSC: Head and Neck Squamous Cell Carcinoma
BLCA: Bladder Urothelial Carcinoma
READ: Rectum Adenocarcinoma
BRCA: Breast Invasive Carcinoma
GBM: Glioblastoma Multiforme
CSPC: castration-sensitive prostate cancer
Mstage: metastasis stage
