# Supplementary material for: Enhancing cancer susceptibility to disulfidptosis by inducing cell cycle arrest and impairing DNA repair
Source: Theranostics. 2026 Jan 1;16(2):637–50. doi: 10.7150/thno.122956 (PMC12674887; doi:10.7150/thno.122956)
Supplement: Supplementary file 1 — Supplementary figures and table legends. [file thnov16p0637s1.pdf]

Supplementary information of

Enhancing cancer susceptibility to disulfidptosis by inducing cell cycle arrest and impairing DNA repair

Jing Lin[1, *], Xueli Yang[2,3,4, *], Cizhong Jiang[5, ✉], Xiaoguang Liu[2,3,4, ✉], Jiejun Shi[1, ✉]

[1] Key Laboratory of Spine and Spinal Cord Injury Repair and Regeneration of Ministry of Education, Tongji Hospital affiliated to Tongji University, Frontier Science Center for Stem Cell Research, School of Life Sciences and Technology, Tongji University, Shanghai 200092, China

[2] Cancer Institute (Key Laboratory of Cancer Prevention and Intervention, China National Ministry of Education) of the Second Affiliated Hospital and Institute of Translational Medicine, Zhejiang University School of Medicine, Hangzhou, China

[3] Cancer Center of Zhejiang University, Hangzhou, China

[4] Zhejiang Key Laboratory of Frontier Medical Research on Cancer Metabolism

[5] Shanghai Tenth People's Hospital, Shanghai Key Laboratory of Signaling and Disease Research, School of Life Sciences and Technology, Tongji University, Shanghai 200072, China

✉ To whom correspondence should be addressed. E-mails: czjiang@tongji.edu.cn, xiaoguangliu@zju.edu.cn, shij@tongji.edu.cn

* The first two authors contributed equally to this work.

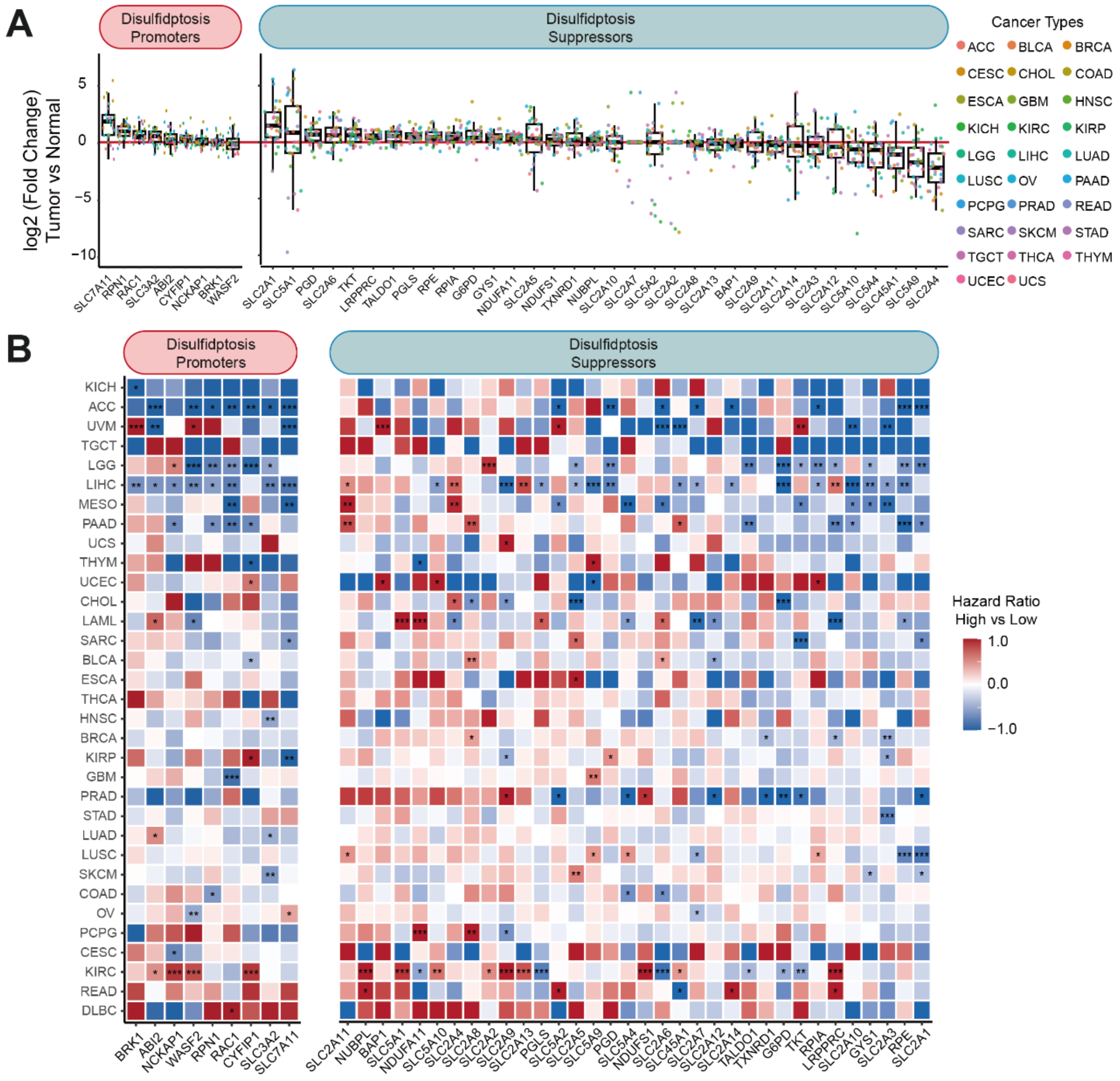

Figure S1. (A) Gene expression differences between tumor and normal tissues for disulfidptosis promoters and suppressors. (B) Prognostic significance of disulfidptosis promoters and suppressors across different cancer types.

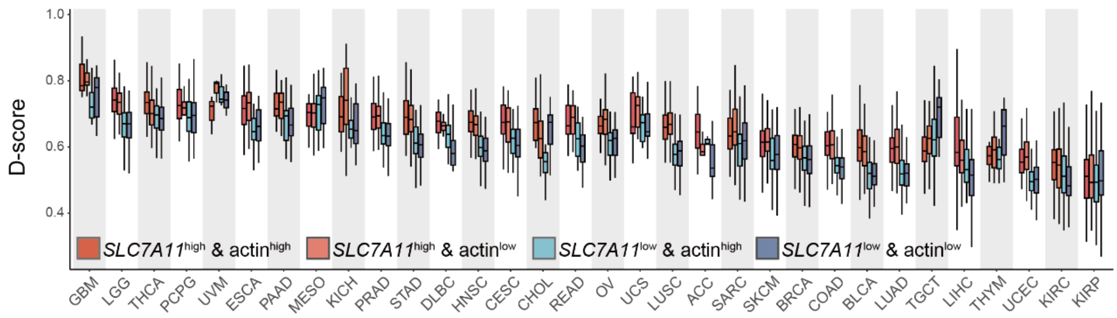

Figure S2. Comparison of D-scores across four patient groups in TCGA cancer types: *SLC7A11*^{high} & actin^{high}, *SLC7A11*^{high} & actin^{low}, *SLC7A11*^{low} & actin^{high}, and *SLC7A11*^{low} & actin^{low} (related to Fig. 1D).

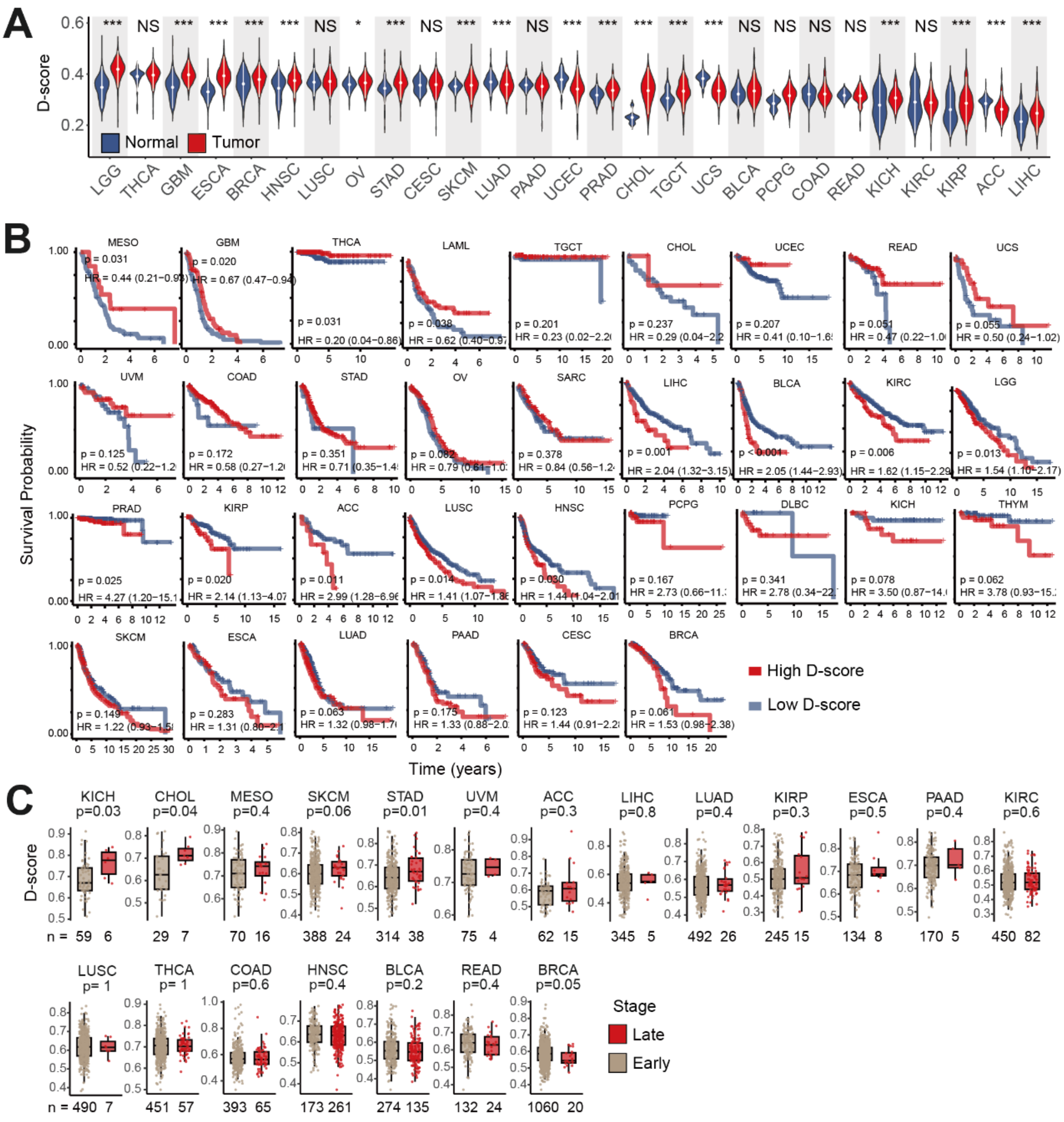

Figure S3. Pan-cancer disulfidptosis susceptibility and its prognostic relevance. (A) Disulfidptosis susceptibility is generally higher in tumor samples compared to matched normal tissues across most cancer types. Statistical significance was assessed using unpaired two-tailed Wilcoxon test. (B) Kaplan-Meier curves showing the survival impact of D-score. P-values are determined by log-rank test. (C) Comparison of D-scores between early-stage and late-stage cancer patients across cancer types. Sample sizes are indicated below each box. Statistical significance was assessed using unpaired two-tailed Student's t-test.

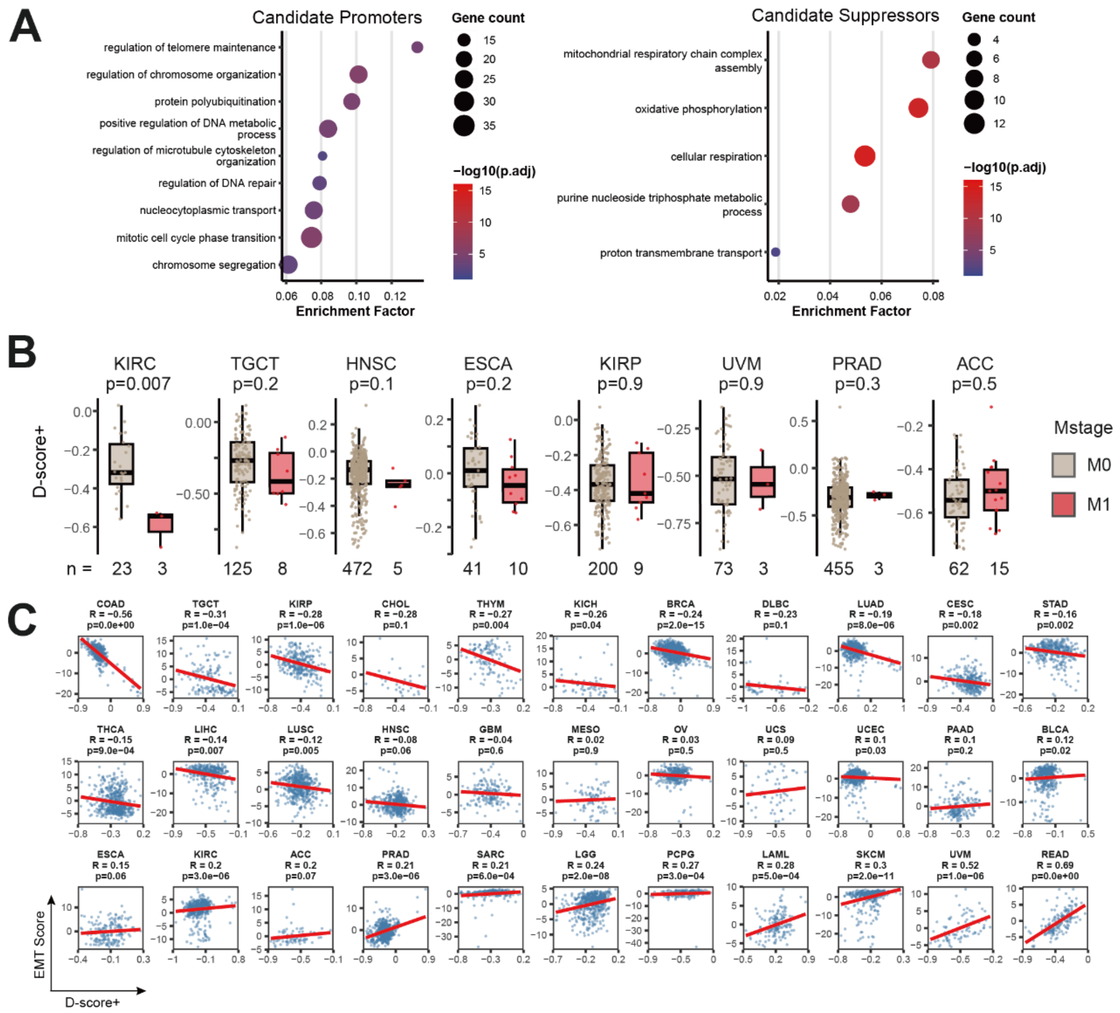

Figure S4. (A) GO functional enrichment analysis of candidate promoters and suppressors. (B) Comparison of D-score+ between patients with (M1, in red) and without (M0, in grey) distant metastasis. Sample sizes are indicated below each box. Statistical significance was assessed using unpaired two-tailed Student's t-test. (C) Spearman's correlation between D-score+ and EMT activity.

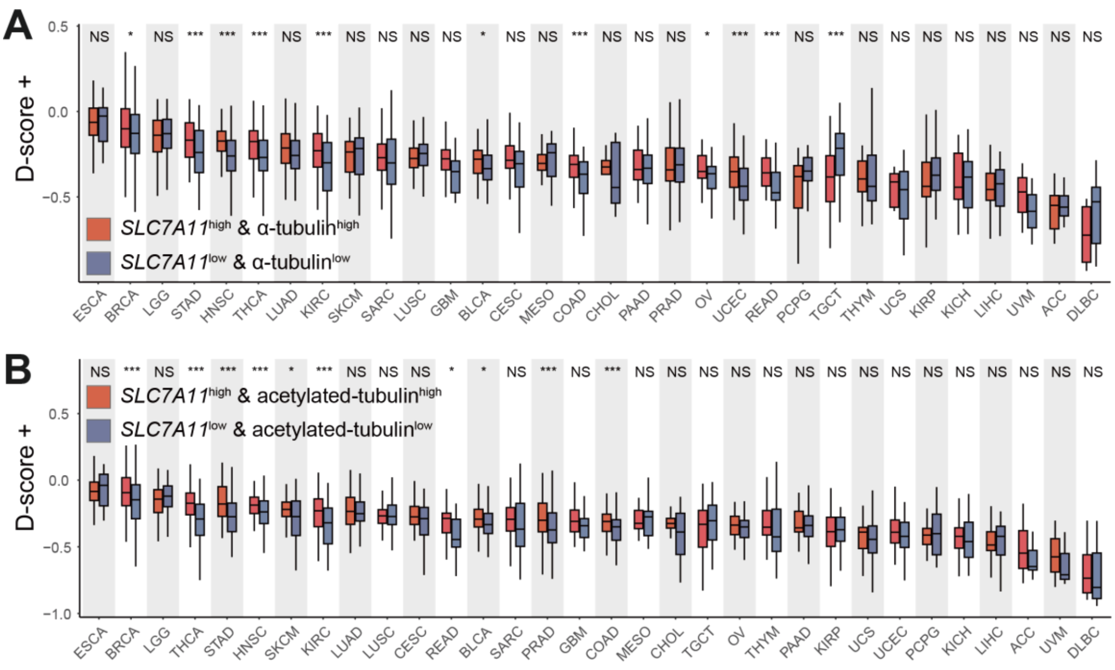

Figure S5. (A) Higher D-score+ in *SLC7A11*^{high} & α-tubulin^{high} group compared to the *SLC7A11*^{low} & α-tubulin^{low} group. (B) Higher D-score+ in the *SLC7A11*^{high} & acetylated-tubulin^{high} group compared to the *SLC7A11*^{low} & acetylated-tubulin^{low} group.

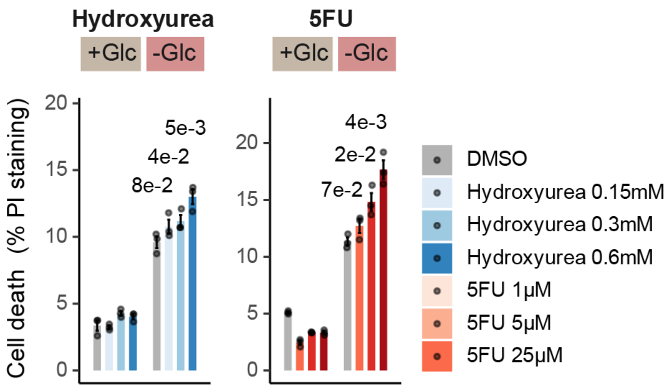

Figure S6. Cell cycle arrest drugs enhance cell death in disulfidptosis cell models of KYSE-150. Cell death was quantified in three replicates. Statistical significance between untreated (DMSO) and treated samples of each drug concentration was assessed using unpaired one-tailed Student's t-test.

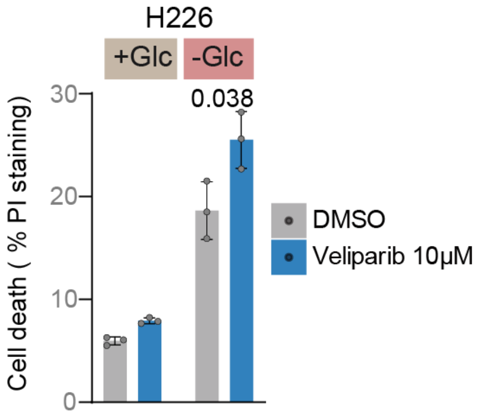

Figure S7. Synergistic effects between disulfidptosis and Veliparib in NCI-H226 lung cancer cell line. Cell death was quantified in three replicates. Statistical significance between untreated (DMSO) and treated samples was assessed using unpaired one-tailed Student's t-test.

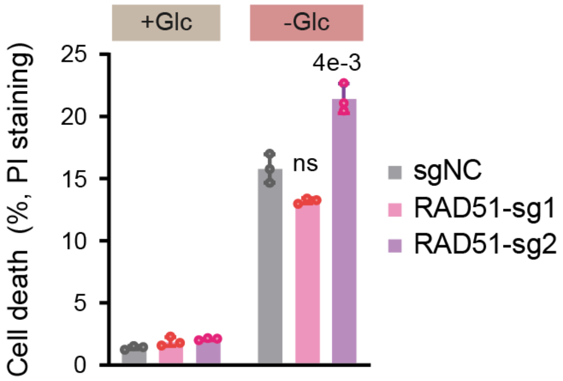

Figure S8. LOVO cells expressing control sgRNA (sgNC) or sgRNAs targeting *RAD51* were cultured in glucose-replete (+Glc) or glucose-starved (-Glc) conditions. Cell death was measured in 3 replicates. Statistical significance was assessed using unpaired one-tailed Student's t-test.

Table S1. Lists of known disulfidptosis promoters(n=9) and suppressors(n=34) identified by previous studies.

Table S2. Lists of candidate disulfidptosis promoters(n=475) and suppressors(n=31) identified by correlation-based screening in this study.
